# Validating anti-inflammatory and cytotoxic properties of *Fagonia cretica* L. through metabolic, in vitro, and in silico profiling

**DOI:** 10.1186/s12906-024-04684-y

**Published:** 2024-11-28

**Authors:** Enas I. A. Mohamed, Ahlam H. Elwekeel, Dalia El Amir Mohamed, Mohamed A. Zaki, Marwa H. A. Hassan

**Affiliations:** Department of Pharmacognosy, Faculty of Pharmacy, Beni-Suef University, Beni-Suef, 62514 Egypt

**Keywords:** *Fagonia cretica*, Cytotoxicity, Anti-inflammatory, Antioxidants, LC-HRMS, Docking

## Abstract

**Background:**

*Fagonia cretica* L. (Family: Zygophyllaceae), is a wild shrub mostly found in Mediterranean districts and extensively used in folk medicine for a vast array of purposes such as antidiabetic and anticancer during the early stages. The goal of the current study was to validate the antioxidant, anti-inflammatory, and cytotoxic properties of Egyptian *F. cretica* using in vitro studies, metabolic profiling, and in silico approaches.

**Methods:**

The plant was collected from the Egyptian desert and the alcoholic extract was prepared from its aerial parts, total phenolic and total flavonoid contents were evaluated spectrophotometrically. Antioxidant potential was assessed *via* 1,1 diphenyl-2-picrylhydrazyl (DPPH) scavenging activity. Anti-inflammatory activity was validated through in vitro COX-2, COX-1, and nitric oxide inhibition. Cytotoxicity was tested against liver (HepG2), breast (MCF-7), and intestinal (CACO2) carcinoma cell lines followed by assessment of its impact on the levels of apoptotic markers namely topoisomerase I and caspase 9 enzymes. Chemical profiling of the extract was performed using LC-HRMS technique. Saponin rich extract was prepared and tested for affecting topo I and caspase 9 enzymes. In silico studies were conducted on anti-inflammatory (COX-2 and COX-1) and cytotoxicity (topoisomerases I, II*α*, and II*β*) targets using Autodock vina in PyRx platform.

**Results:**

Total phenolic and total flavonoid content of the extract were 2.4 ± 0.12 mg GAE/g and 0.18 ± 0.01 mg RE/g, respectively. In vitro results revealed antioxidant activity calculated as 1.4 ± 0.1 mg AEAC/g. In vitro anti-inflammatory assays unveiled inhibition of COX-2 and COX-1 enzymes with IC_50_ values of 13.02 ± 0.61 and 26.51 ± 0.83 µg/ml, respectively and nitric oxide with IC_50_ of 147.05 ± 9.61 µg/ml. Cytotoxicity on MCF-7, HepG2, and CACO2 cell line with IC_50_ values of 6.9 ± 0.53, 7.6 ± 0.42, and 9.2 ± 0.35 µg/ml, respectively, in addition to in vitro topoisomerase I inhibition (IC_50_ = 13.57 ± 0.71 µg/ml) and caspase 9 induction by 5.66 folds. Metabolic profiling using LC-HRMS technique resulted in dereplication of 21 compounds including triterpenoid saponins, flavonoids, diterpenoids, etc. Interestingly, saponin rich fraction and non-saponin fraction exhibited similar effects on topoisomerase I and caspase 9. In silico investigation unveiled high binding affinities of almost all the detected metabolites to the active sites of COX-2, COX-1, topo I, II*α*, and II*β* enzymes.

**Conclusion:**

Collectively, we can conclude that *F. cretica* is a new source of many phytochemicals, and a significant natural source as cytotoxic and anti-inflammatory agent.

## Introduction

Recently, natural herbs have received a great deal of attention throughout the whole world for the discovery of biologically active substances. Many studies have focused on medicinal plants rich in secondary metabolites or phytopharmaceuticals which exhibit potent pharmacological activities, such as antibacterial, antiviral, antioxidants, anti-inflammatory, antimutagenic, and cytotoxic effects [1].

The desert regions in Egypt comprise about 90% from its geographical area, many wild plants are growing in these areas and unexplored until now. These plants can produce a wide range of chemical substances that help them execute crucial biological processes. When consumed by humans, several of these plants have long-term health benefits and can be utilized to cure human ailments effectively [2]. *Fagonia* is a genus within the Zygophyllaceae family, comprising 34 species [3], about 15 to 18 species are found in Egypt [4, 5]. Traditionally, various species of this genus are used as aqueous or alcoholic tincture for treatment of diabetes, fever, asthma, toothache, stomach pain and kidney problems [6]. *Fagonia cretica* (L) is one of the notable species which is distributed in the Egyptian deserts and is known for its high content of saponins and flavonoids [7]. It is a spiny branched shrub with compound leaves of three to five leaflets, small white to pale pink flowers, capsule fruit containing small brown to black seeds [8]. Traditionally, *F. cretica* is considered as a cornerstone of folk medicine in many parts of the world, an aqueous decoction of *F. cretica* aerial parts was used to control the early stages of cancer, as well as digestive and cardiovascular disorders [9]. In folk medicine, *F. cretica* has long been attributed with its antidiabetic properties [10]. Moreover, studies on *F. cretica* have demonstrated many pharmacological activities such as antimicrobial [11], anti-inflammatory [12] and cytotoxicity activities [13].

Dereplication technique becomes one of the most important lines in drug discovery. Dereplication improves and speeds up the drug discovery efficiency through quick identification of known secondary metabolites that saves efforts for the discovery of new metabolites [14]. Dereplication could be used as an untargeted method for identification of the main plant metabolites or for speeding up bioactivity guided fractionation process. In another way, dereplication is combined with metabolic researches to perform untargeted chemical profiling of natural extracts or to identify a specific class of metabolites [15].

Herein, our study aimed to biological potential investigation of Egyptian *F. cretica* including in vitro antioxidant, anti-inflammatory, and cytotoxic activity together with measuring the impact on the levels of apoptotic markers using topoisomerase I (topo I) and caspase-9 (casp-9) assays. In addition to conducting metabolic analysis of the plant using LC-HRMS, in silico studies were also employed to explore the potential mechanisms behind its biological activities.

## Materials and methods

### Collection of the plant

The aerial parts of naturally occurring *F. cretica* L. (Family: Zygophyllaceae) were gathered from the Mediterranean coast of Egypt in March 2017. Collected plant specimens were identified and authenticated by Dr. Ibrahim El-Garf, Faculty of Science, Cairo University and Dr. Abdel Haleem Mohamed, Flora and Phyto-taxonomy Department, Agriculture Research Center, Giza, Egypt. A voucher specimen (MZ/1) was deposited at the herbarium of Faculty of Science, Cairo University. The plant material was air-dried and grounded into powder. The grinded plant was maintained in an airtight container and kept for further analysis.

### Chemicals and solvents

Methanol (MeOH) were purchased from El-Nasr Company for Pharmaceuticals and Chemicals (Egypt). Solvents for High Performance Liquid Chromatography (HPLC) were purchased from Sigma-Aldrich (Saint Louis, Missouri, USA), including acetonitrile and water. For the biological study, DPPH, rutin and ascorbic acid were purchased from Sigma-Aldrich (Saint Louis, Missouri, USA).

### Preparation of the extract for total phenolic (TPC) and flavonoid contents (TFC) and antioxidant activity

One gram of the air-dried powdered plant was extracted using 50 ml methanol (80%) for 2 h at room temperature on an orbital shaker adjusted at 200 rpm. After centrifugation for 20 min the supernatant was transferred to a 100 ml volumetric flask. The procedure was repeated, and the collective supernatant volume was adjusted to 100 ml.

### Total phenolic content (TPC)

TPC was measured using Folin-Ciocalteu reagent. Alcoholic extract (300 µl) was added to 2.25 ml Folin-Ciocalteu reagent, allowed to stand for 5 min at room temperature then 2.25 ml of sodium carbonate solution (60 g/L) was added, and the mixture was incubated for 90 min at room temperature. The absorbance of the developed color was determined by spectrophotometer at 725 nm. Gallic acid was used to prepare the standard curve for quantitative purposes, where the result was calculated as mg of gallic acid/g dry plant powder (GAE/g).

### Total flavonoid content (TFC)

It was evaluated spectrophotometrically using slightly modified method of [16]. In a test tube, 0.5 ml of the alcoholic extract was mixed with 2.25 ml distilled water and 0.15 ml NaNO_2_ solution. The tubes were left for 6 min, then 0.3 ml AlCl_3_.6H_2_O solution (10%) was added and allowed to stand for 5 min followed by addition of 1.0 ml NaOH (1 M). The mixture was mixed well using vortex, and the absorbance was immediately measured at 510 nm. The result was expressed as mg rutin equivalents for 1 g dried sample (mg RE/g).

### Antioxidant activity

The scavenging activity of the methanol extract was evaluated using 1,1 diphenyl-2-picrylhydrazyl (DPPH) as a free radical model [17]. An aliquot of 300 µl of the sample was mixed with 3.0 ml of 250 μm DPPH in absolute ethanol and the mixture was vigorously shaken and allowed standing for 30 min in the dark at 25 °C. The absorbance was measured spectrophotometrically at 517 nm, using ascorbic acid as positive control. The free radical scavenging activity was calculated from the following equation:

Scavenging effect (%) = [1-(absorbance of sample /absorbance of control)] × 100.

The result of antioxidant activity was expressed as mg of ascorbic acid/g dry plant powder (AEAC/g).

### Anti-inflammatory activity

#### Cyclooxygenase inhibitory activity (COX-2 and COX-1)

The COX-2 and COX-1 inhibitory activity of the alcoholic extract in addition to celecoxib and indomethacin, as reference standards were measured in vitro by enzyme immunoassay (EIA) human COX-2 and COX-1 inhibitor screening kit according to the manufactures protocol (catalog number 701070 and 701080, respectively, Cayman Chemical, Ann Arbor, MI, USA) [18, 19]. The samples to be tested were dissolved in DMSO (Sigma, St. Louis, Mo., USA) and several concentrations were incubated for 10 min at 37 °C with each of COX-2 and COX-1 enzyme, separately, in addition to Heme in the supplied reaction buffer (0.1 M Tris-HCl pH 8 containing 5 mM EDTA and 2 mM phenol). Then, 100 mM arachidonic acid was added to begin the reaction, and it was stopped immediately after 30 s by addition of stannous chloride. This was followed by the quantification of PGF2α (Prostaglandin F2α) formed in the samples by EIA (enzyme immunoassay). The optical density was determined after 1 h using a microplate reader DYNATech (Dynatech Microplate Reader), MR 5000 (Dynatech Industries Inc., McLean, VA, USA) at 450 nm. The percentage of inhibition was calculated for the different concentrations tested against the control, and the IC_50_ values against both COX-2 and COX-1 enzymes were calculated from the concentration inhibition response curve.

#### Nitric oxide inhibitory assay (Griess nitrite assay)

RAW 264.7 murine macrophage cells were stimulated by LPS (Lipopolysaccharide) to produce inflammatory mediators as NO, the produced nitrite (NO_2_-) was measured using the Griess reagent [20], RAW 264.7 murine macrophage-like cells were grown in phenol red-free RPMI-1640 containing 10% FBS. Cells were incubated for 24 h with LPS (1 mg/ml) in the presence of different concentrations of the plant extract. Then 50 µl of supernatant from each well of cell culture was transferred into 96-well microplates and equal volume of Griess reagent (1% sulfanilamide, 0.1% *N*-(1-naphthyl)-ethylenediamine hydrochloride, 2.5% H_3_PO_4_) was then added to the supernatant at room temperature. The absorbance at 550 nm was determined in UV-Vis microplate reader after 10 min. The concentrations of nitrite were derived from regression analysis using serial dilutions of sodium nitrite as a standard. Percentage of inhibition was calculated based on the ability of the extract to inhibit nitric oxide formation by cells compared with the control (cells in the media without the extract containing triggering agents and DMSO), which was considered as 0% inhibition.

### Cytotoxic activity

The Cytotoxic activity was tested using the viability assay following the method reported by Mosmann, T., 1983 [21, 22]. HepG2 cells (liver carcinoma cell line), MCF-7cells (breast carcinoma cell line) and CACO2 cells (intestinal carcinoma cell line) (American Type Culture Collection ATCC, Rockville, MD) were applied through the tissue Culture Unit, the Egyptian Organization for Biological Products and Vaccines, Vacsera, Egypt. In brief, the tumor cell lines were suspended in medium at concentration 5 × 10^4^ cell/ well in Corning^®^ 96-well tissue culture plates and then incubated for 24 h. The alcoholic extract of *F. cretica* was then added into 96-well plates (three replicates) to achieve twelve concentrations. Six vehicle controls with media or 0.5% DMSO were run for each 96 well plate as a control. After incubating for 24 h, the number of viable cells was determined by the MTT test. Briefly, the media was removed from the 96 well plates and replaced with 100 µl of fresh culture RPMI 1640 medium without phenol red then 10 µl of the 12 mM MTT stock solution (5 mg of MTT in 1 ml of PBS) to each well including the untreated controls. The 96 well plates were then incubated at 37 °C and 5% CO_2_ for 4 hours. An 85 µl aliquot of the media was removed from the wells, and 50 µl of DMSO was added to each well and mixed thoroughly with the pipette and incubated at 37 °C for 10 min. Then, the optical density (OD) was measured at 590 nm with the microplate reader (SunRise, TECAN, Inc, USA) to determine the number of viable cells and the percentage of viability was calculated as follows:

Viability= [OD (treated cells)/ OD (control cells)] x 100%.

The relation between surviving cells and drug concentration was plotted to get the survival curve of each tumor cell line after treatment with the sample. The IC_50_ value was defined as the concentration of the sample required to inhibit 50% of the cell growth, it was estimated from graphic plots of the dose response curve for each conc. using Graphpad Prism software (San Diego, CA. USA). Doxorubicin was used as positive control. Fetal Bovine serum, DMEM, RPMI-1640, HEPES buffer solution, L-glutamine, gentamycin and 0.25% Trypsin-EDTA were purchased from Lonza.

### Topoisomerase I inhibitory assay

To measure topo I levels, Human TOP1 (DNA topoisomerase 1) ELISA kits (Catalog No.: MBS763851) were used. Briefly, the plate was washed 2 times before adding standard, sample (diluted at least 1/2 with sample dilution buffer) and control (blank) wells. The standard or sample were added 100 µl to each well and incubated for 90 min at 37 °C. Plates were washed 2 times. Then 100 µl Biotin-labeled antibody working solution was added to each well and inoculated at 37 °C for 60 min followed by washing the plates 3 times. The SABC (HRP-Streptavidin Conjugate) working solution 100 µl was added to each well and incubated for 30 min at 37 °C, then the plates were washed 5 times. TMB (3,30,5,50-Tetramethylbenzidine) substrate solution 90 µl was added and incubated at 37 °C for 10–20 min. Finally, the stop solution 50 µl was added and the absorbance was recorded at 450 nm immediately using spectrophotometer [23].

### Effect on the levels of caspase-9 enzyme

To measure caspase-9 levels, DRG^®^ Caspase-9 (human) ELISA (EIA-4860) kits were used. In brief, the breast cancer cell line (MCF-7) extracts were prepared after elevation of apoptosis. The extracts were suspended in lysis buffer, and incubated at room temperature for 60 min and then centrifuge for 15 min. The microwell strips were washed with 400 µl wash buffer, followed by addition of 100 µl of sample diluent in duplicate to all standard wells, 100 µl of sample diluent in duplicate to the blank wells, 50 µl of sample diluent to the sample, and 50 µl of each sample in duplicate to the sample wells. Then the detection antibody 50 µl was added to all wells and incubated for 2 h at room temperature. The diluted antirabbit-IgG-HRP (anti-rabbit-immunoglobulin G-horseradish peroxidase) 100 µl was added to all wells together with blank wells. TMB substrate solution 100 µl was added to all wells and incubated for 15 min. at room temperature. Finally, the enzyme reaction was stopped by adding 100 µl of the stop solution to all wells. The absorbance of each microwell was measured at 450 nm using a spectrophotometer. The absorbance of the samples and standard were recorded [24, 25].

### Metabolic profiling

Dereplication or chemical profiling of the crude alcoholic extract of *F. cretica* was carried out using LC-HRMS technique [26]. An ACQUITY Ultra Performance Liquid Chromatography system coupled with a Synapt G2-HDMS quadrupole-time-of-flight hybrid mass spectrometer (Waters, Milford, MA, USA) was used for this analysis. The column for UPLC was on an ethylene bridged hybrid (BEH) C_18_ column (2.1 × 100 mm, particle size 1.7 μm; Waters, Milford, MA, USA) with a guard column (2.1 × 5 mm, particle size 1.7 μm) and a linear binary solvent gradient of 0–100% eluent B over 6 min at a flow rate of 0.3 ml/min^− 1^ using 0.1% formic acid in water (v/v) as solvent A and acetonitrile as solvent B. The injection volume was 2 µl and the column temperature was 40 °C. Electrospray ionization (ESI) in the positive mode was used and the source was operated at 120 °C. The ESI capillary voltage was set to 0.8 kV, the sampling cone voltage was set to 25 V, and nitrogen (at 350 °C, flow rate (FR) 800 L/h) was used as the de-solvation gas and the cone gas (FR 30 L/h). The mass range for the TOF–MS was set according to the mass-to-charge ratio (*m/z*) 50–1,200. The raw data files were then submitted to the data chromatogram builder in the software MZmine 2.12 (Okinawa Institute of Science and Technology Graduate University, Japan), that was used to detect the peaks. Mass ion peaks were separated using the following steps: raw data was imported by selecting the ProteoWizard-converted positive files in the *mz* ML format, then the ions were detected, followed by a chromatogram builder and a chromatogram deconvolution. The local minimum search algorithm was applied, and isotopes were also identified via the isotopic peaks’ grouper. The missing peaks were detected using a gap-filling peak finder. An adduct search as well as a complex search were performed. The processed data set was then subjected to molecular formula prediction and peak identification. The positive data set from the respective plant extract was dereplicated against the Dictionary of Natural Products (DNP) database.

### Preparation of saponin-rich fraction

The saponin-rich fraction was prepared using cold acetone method [27]. Five grams of the alcoholic extract were dissolved in 20 ml of 70% ethanol and then partitioned with 50 ml of *n*-hexane three times. The resulting fractions were evaporated under reduced pressure, yielding 0.86 g of the *n*-hexane fraction and 3.55 g of the alcoholic fraction. The alcoholic fraction was then resuspended in 20 ml of 70% ethanol, and cold acetone was added drop by drop, up to 100 ml. The phytoconstituents with low solubility in cold acetone were precipitated. The precipitated phytoconstituents were separated and both the precipitated constituents (saponin-rich fraction) and the soluble constituents (non-saponin fraction) were dried under vacuum to yield (1.23 g) and (1.48 g), respectively. Both fractions were assigned for investigating their effect on the levels of apoptotic markers namely topoisomerase I and caspase 9 enzymes.

### Molecular docking

Structures of the detected metabolites were downloaded from PubChem database [28]. Crystal structures of topo I (1EJ9) [29], topo II*α* (5GWK) [30], topo II*β* (3QX3) [31], COX-2 (5IKV) [32], and COX-1 (6Y3C) [33], were retrieved from the protein data bank https://www.rcsb.org/. For each protein, water molecules were removed, polar hydrogens and kollman charges were added during protein preparation step. Docking studies were conducted using PyRx, Autodock vina software where the grid box for each enzyme was located to clarify the binding site. XYZ coordinates were set as: 1EJ9: 1.00, 3.11, 31.67; 5GWK: 16.67, -42.45, -50.01; 3QX3: 37.08, 90.00, 59.01; 5IKV: 167.72, 184.20, 187.19; 6Y3C: -33.21, -43.59, 8.54. Docking outputs were visualized using Pymol molecular graphics system version 2.5.5 [34] and Discovery studio visualizer v21.1.0.20298 (Dassault systems Biovia Corp., San Diego, CA, USA).

### Statistical analysis

All the results of the anticancer, antioxidant, and antiinflammatory activities together with phenolics and flavonoids assay are expressed as mean values ± SD from three separate experiments. The IC_50_ values were calculated from the dose response curves using non-linear regression analysis that gave a percentage of the inhibition values.

## Results

### Total phenolic (TPC) and total flavonoid (TFC) contents and antioxidant activity

TPC and TFC of *F. cretica* were ascertained to be 2.4 mg ± 0.12 GAE/g and 0.18 ± 0.01 mg RE/g, respectively, while the antioxidant activity determined by DPPH radical scavenging assay was 1.4 ± 0.1 mg AEAC/g (Table 1).

**Table 1:** Total phenolic (TPC) and flavonoid (TFC) contents and antioxidant activity of the alcoholic extract of *F. cretica* L. aerial parts

Extract	Phenolic content (mg GAE/g)^a^	Flavonoid content (mg RE/g)^b^	Antioxidant activity (mg AEAC/g)^c^
*Fagonia cretica*	2.4 ± 0.12	0.18 ± 0.03	1.40 ± 0.1

^a^ mg gallic acid equivalent in 1 g of dry sample, ^b^ mg rutin equivalent in 1 g of dry sample, ^c^ mg ascorbic acid equivalent antioxidant capacity (AEAC) in 1 g of dry sample

### Anti-inflammatory activity

Two parameters were utilized to evaluate the anti-inflammatory activity: cyclooxygenase (COX) enzymes inhibition and nitric oxide (NO) inhibition. Results suggested that the alcoholic extract of *F. cretica* produced COX-2 and COX-1 inhibition with IC_50_ values of 13.02 ± 0.61 and 26.51 ± 0.83 µg/ml, respectively compared to celecoxib and indomethacin (2.79 ± 0.14 and 1.25 ± 0.07 µg/ml, respectively). On the other hand, applying modified Griess method [35] to the plant extract revealed that it inhibited NO with IC_50_ (147.05 ± 9.61 µg/ml) compared to ascorbic acid (18.73 ± 0.77 µg/ml) (Table 2).

**Table 2:** Anti-inflammatory activity of the alcoholic extract of *F. cretica* L. aerial parts against COX-1, COX-2 and NO

Extract	IC_50_ (µg/ml)		
	COX-1	COX-2	NO
*Fagonia cretica*	26.51 ± 0.83	13.02 ± 0.61	147.05 ± 9.61
Celecoxib	91.75 ± 6.04	2.79 ± 0.14	NA
Indomethacin	1.25 ± 0.07	172.93 ± 6.53	NA
Ascorbic acid	NA	NA	18.73 ± 0.77

NA: not applicable

### Cytotoxic activity

#### Anticancer activity using MTT assay

The results in (Table 3) showed that the extract exhibited significant inhibitory activity against MCF-7 cell line with IC_50_ = 6.9 ± 0.53 µg/ml, which is significantly lower than that of doxorubicin with IC_50_ = 9.15 ± 0.5 at (*p* < 0.01). Also, the extract showed an inhibitory effect against HepG2 with IC_50_ = 7.6 ± 0.42 µg/ml and CACO2 with IC_50_ = 9.2 ± 0.35 µg/ml which were significantly higher than that of with IC_50_ = 6.28 ± 0.4, and 7.75 ± 0.4 µg/ml at (*p* < 0.05).

**Table 3:** In vitro anticancer activity of the alcoholic extract of *F. cretica* L. aerial parts against three human carcinoma cell lines

Extract	IC_50_ (µg/ml)		
	MCF-7	HepG2	CACO2
*Fagonia cretica*	6.9 ± 0.53**	7.6 ± 0.42*	9.2 ± 0.35*
Doxorubicin	9.15 ± 0.50	6.28 ± 0.40	7.75 ± 0.40

**HepG2**: Liver carcinoma cell lines, **MCF-7**: Breast carcinoma cell lines, **CACO2**: Intestinal carcinoma cell line

The results are expressed as Mean ± S.D, they were processed adopting student t-test

** significant versus doxorubicin at *p* < 0.01

* significant versus doxorubicin at *p* < 0.05

### The impact on the levels of apoptotic markers

In Table 4 results showed that the alcoholic extract inhibited Topo I with IC_50_ value of 13.57 ± 0.71 µg/ml compared to doxorubicin (IC_50_ = 5.755 ± 0.3 µg/ml) and induced caspase-9 level by 5.66 folds compared to doxorubicin which posted caspase-9 level with 8.67 folds.

**Table 4:** Results of topoisomerase I and caspase 9 assays for the total alcoholic extract, saponin-rich fraction, and non-saponin fraction of *F. cretica* L. aerial parts using breast human carcinoma cell lines

Extract	Caspase-9 (Conc., ng/ml)	Topoisomerase-1 (IC_50_, µg/ml)
*Fagonia cretica* (Total extract)	16.4 ± 0.81	13.57 ± 0.71
Saponin-rich fraction	11.48 ± 0.23	15.73 ± 0.24
Non-saponin fraction	14.84 ± 0.27	11.91 ± 0.71
Doxorubicin	25.84 ± 0.39	5.755 ± 0.3
Control	2.986 ± 0.31	NA

NA: not applicable

#### Metabolic profiling

Metabolic profiling of the phytoconstituents from the collected desert plant *F. cretica* using LC-HRMS technique resulted in dereplication of 21 compounds (Table 5). The interpreted compounds comprise a wide variety of constituents such as flavonoids, diterpenes, sterols, and triterpenes. Twenty-one compounds were identified as one fatty acid linoleic acid (1), previously detected in *F. cretica*. One pterocarpan (isoflavonoid derivative) atricarpan B (4); is the first time to be detected in *Fagonia* species. Four flavonoids; isorhamnetin 3,7-diglucoside (2), isorhamnetin (10) or sexangularetin (11), and isorhamnetin-*α*-3-*O*-L-rhamnoside (17), recorded for the first time in *F. cretica* species together with rutin (20). Two sulfur containing triterpenes; 3*β*-(2-*O*-sulfo-R-L-arabinopyranosyl)-27-dihydroxyurs-12-en-28-oic acid 28-*O*-*β*-D-glucopyranoside (7) and 3-*O*-([*β*-D-4-*O*-sulfonylglucopyranosyl-(l-3)]-[*β*-D-xylopyranosyl-(l-2)]-*α*-L-arabinopyranosyl)-ursolic acid-28-*O*-[*β*-D-glucopyranosyl] ester (indicasaponin D) (12), detected for the first time in *F. cretica* species. Other eight triterpene saponins were identified as 3-*O*-[*β*-D-glucopyranosyl (1–2)-*α*-L-arabinopyranosyl]-27-hydroxy oleanolic acid 28-O-[*β*-D-glucopyranosyl (1–6)-*β*-D-glucopyranosyl] ester (6), oleanolic acid (9), oleanolic aldehyde acetate (13), oleanolic acid 3-*O*-*α*-L-rhamnopyranosyl (1–3)-6'-*O*-methyl-*β*-D-glucuronopyranosyl 28-*O*-*β*-D-glucopyranoside (14), 22-hydroxyursolic acid (15), quinovic acid 3-*O*-*β*-D-glucopyranoside (16), 3-*O*-D-glucopyranosyl-(1→3)-*α*-L-arabinopyranoside oleanolic acid (19) and 3*β*-*O*-[*β*-D-glucopyranosyl (1→2)-*α*-L-arabinopyranosyl] olean-12-en-27-al-28-oic acid 28-*O*-[*β*-D-glucopyranosyl (1→6)-*β*-D-glucopyranosyl] ester (21). Three diterpenes; 7*β*-hydroxy-5-epi-fagonene (5), geranyllinalool-3-*O*-*β*-D-glucopyranoside (8), and 16-*O*-acetylfagonone (18) together with one sterol *β*-sitosterol (3), firstly discovered in *F. cretica*.

**Table 5:** The LC-HRMS dereplication results of the alcoholic extract of *F. cretica* L. aerial parts

*No.*	*Metabolite name*	*Molecular formula*	*Rt (min)*	*m/z*	*Chemical class*	*Reference*
1.	Linoleic acid	C_18_H_32_O_2_	3.5879	280.1368	Fatty acid	[40]
2.	Isorhamentin 3,7-diglucoside*	C_28_H_32_O_17_	3.81750	640.4577	Flavonoid	[48]
3.	*β*-sitosterol	C_29_H_50_O	4.2356	414.2129	Sterol	[49, 50]
4.	Atricarpan B	C_18_H_16_O_7_	4.2389	344.0979	Pterocarpan (Isoflavonoid derivative)	[51]
5.	7*β*-Hydroxy-5-epi-fagonene*	C_20_H_34_O_3_	4.2622	322.0995	Diterpene	[52]
6.	3-*O*-[*β*-D-glucopyranosyl (1–2)-*α*-L-arabinopyranosyl]-27-hydroxy oleanolic acid 28-*O*-[*β*-D-glucopyranosyl (1–6)-*β*-D-glucopyranosyl] ester*	C_53_H_86_O_23_	5.006	1090.6401	Triterpenoid saponin	[54]
7.	3*β*-(2-*O*-sulfo-R-L-arabinopyranosyl)-27-dihydroxyurs-12-en-28-oic acid 28-*O*-*β*-D-glucopyranoside	C_41_H_66_O_16_S	5.1109	846.4536	Triterpenoid saponin	[41, 42]
8.	Geranyllinalool-3-*O*-*β*-D-glucopyranoside	C_26_H_44_O_6_	5.8282	452.3227	Diterpene	[52]
9.	Oleanolic acid	C_30_H_48_O_3_	5.8370	456.2779	Triterpenoid saponin	[40, 45]
10.	Isorhamnetin	C_16_H_12_O_7_	5.8399	316.1950	Flavonoid	[46]
11.	Herbacetin-8-methyl ether (sexangularetin)	C_16_H_12_O_7_	5.8428	316.1967	Flavonoid	[46]
12.	3-*O*-([*β*-D-4-*O*-sulfonylglucopyranosyl-(l-3)]-[*β*-D-xylopyranosyl-(l-2)]-*α*-L-arabinopyranosyl)-ursolic acid-28-*O*-[*β*-D-glucopyranosyl] ester (Indicasaponin D)	C_52_H_84_O_24_S	6.2526	1124.8413	Triterpenoid saponin	[49]
13.	Oleanolic aldehyde acetate	C_32_H_50_O_3_	6.3548	482.3746	Triterpenoid saponin	[40, 45]
14.	Oleanolic acid 3-*O*-*α*-L-rhamnopyranosyl (1–3)-6'-*O*-methyl-*β*-D-glucuronopyranosyl 28-*O*-*β*-D-glucopyranoside	C_49_H_78_O_18_	6.5059	954.6166	Triterpenoid saponin	[44]
15.	22-Hydroxyursolic acid**	C_30_H_48_O_4_	6.5466	472.2744	Triterpenoid saponin	[40, 45]
16.	Quinovic acid 3-*O*-*β*-D-glucopyranoside**	C_36_H_56_O_10_	6.6120	648.3759	Triterpenoid saponin	[10]
17.	Isorhamnetin-*α*-3-*O*-L-rhamnoside***	C_22_H_22_O_11_	6.6919	462.3607	Flavonoid	[48]
18.	16-*O*-acetylfagonone	C_22_H_34_O_4_	6.7157	362.3102	Diterpene (Erythroxan-type diterpene)	[53]
19.	3-*O*-D-glucopyranosyl-(1→3)-*α*-L-arabinopyranoside oleanolic acid**	C_41_H_66_O_12_	6.8062	750.4486	Triterpenoid saponin	[42]
20.	Rutin**	C_27_H_30_O_16_	9.070	610.048	Flavonoid	[47]
21.	3*β*-*O*-[*β*-D-glucopyranosyl (1→2)-*α*-L-arabinopyranosyl] olean-12-en-27-al-28-oic acid 28-*O*-[*β*-D-glucopyranosyl (1→6)-*β*-D-glucopyranosyl] ester*	C_53_H_84_O_23_	9.2861	1088.6292	Triterpenoid saponin	[54]

*These compounds were detected as M + Na, **These compounds were detected as M + 2 H, other compounds have been detected as M + H

### Impact of saponin-rich fraction on apoptotic markers

Both saponin-rich fraction and non-saponin fraction demonstrated similar impact on the apoptotic markers, where they inhibited topo I with IC_50_ values of 15.73 ± 0.24 µg/ml and 11.91 ± 0.71 µg/ml, respectively and induced caspase-9 by 3.84 and 4.96 folds, respectively (Table 4).

#### Molecular docking

**Fig. 1:**
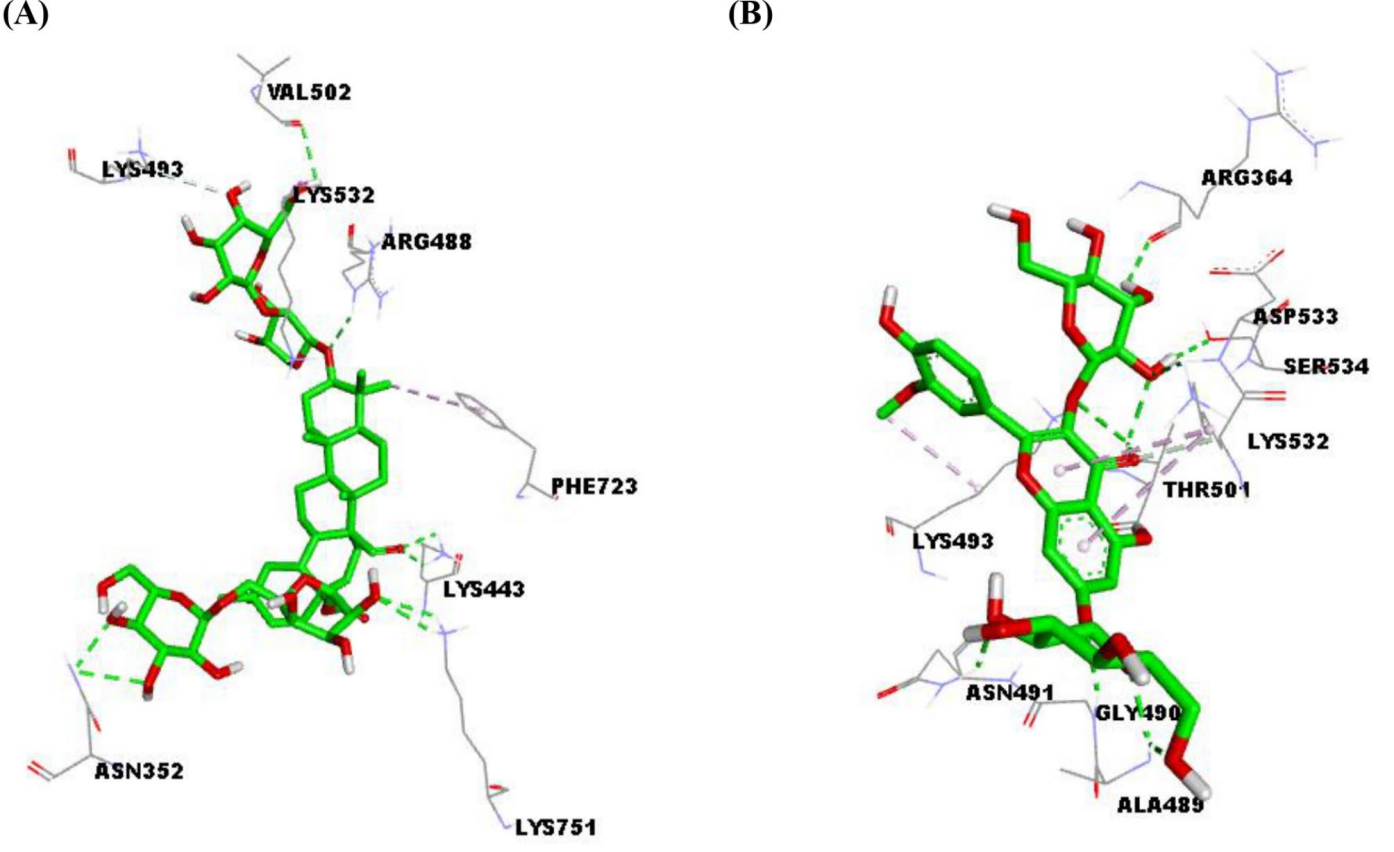
3D depiction of the active site of human topoisomerase I enzyme (1EJ9) showing interacting residues with compound 21 (**A**) and rutin (**B**), ligands are depicted in green stick model and labeled residues in grey line models, and hydrogen bonds are represented by green dashed lines

**Fig. 2:**
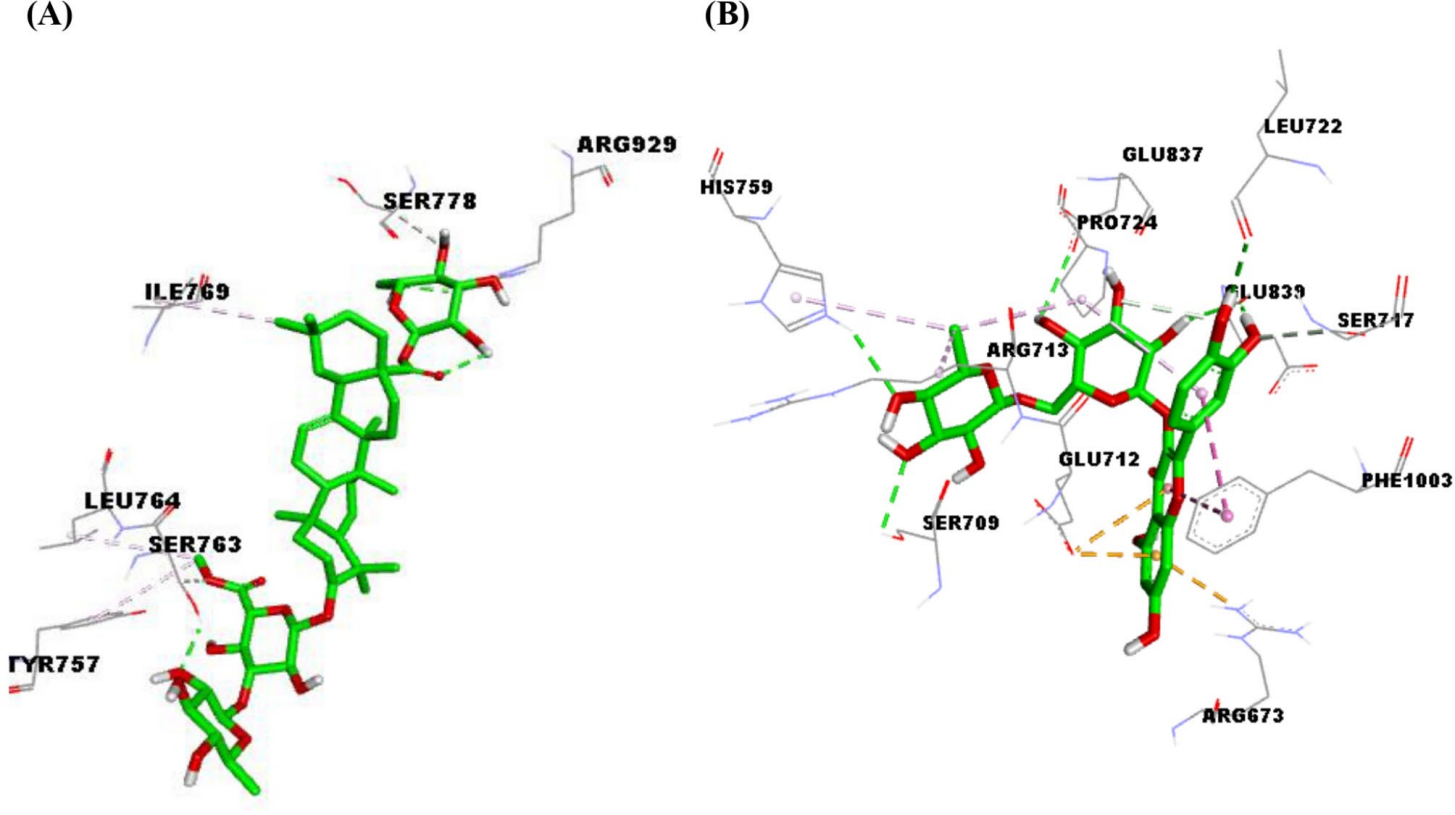
3D depiction of the active site of human topoisomerase II*α* enzyme (5GWK) showing interacting residues with compound 14 (**A**) and rutin (**B**), ligands are depicted in green stick model and labeled residues in grey line models, and hydrogen bonds are represented by green dashed lines

**Fig. 3:**
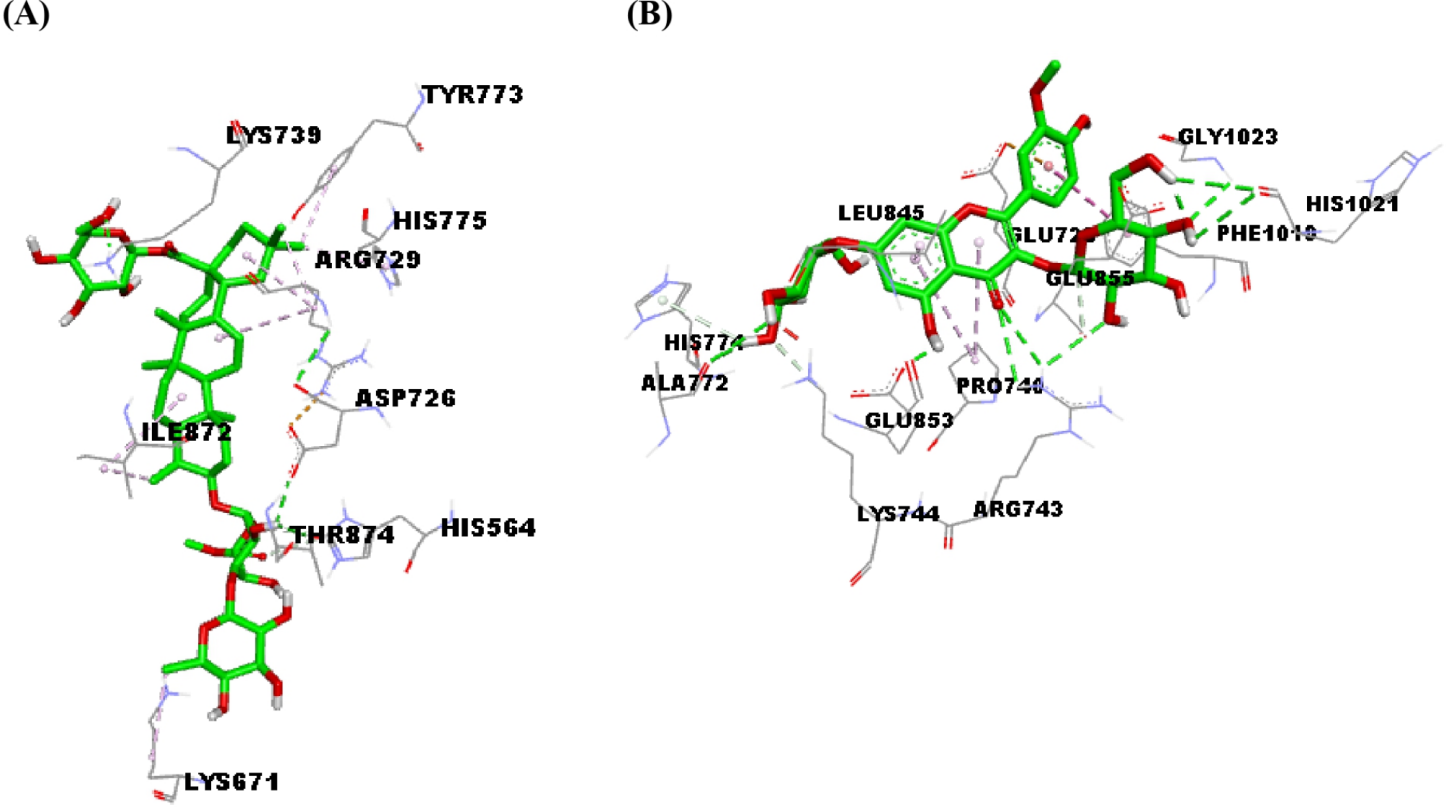
3D depiction of the active site of human topoisomerase II*β* enzyme (3QX3) showing interacting residues with compound 14 (**A**) and isorhametin3,7-diglucoside (**B**), ligands are depicted in green stick model and labeled residues in grey line models, and hydrogen bonds are represented by green dashed lines

The outcomes of docking studies were summarized in (Table 6). Further, 3D depictions representing the interactions of the top-scoring metabolites with the active site residues of each target are shown in (Figs. 1, 2 and 3). Docking of the detected metabolites into the active sites of topo I unveiled binding affinities ranging from -5.8 to -9.5 kcal/mol, while for topo II*α* and topo II*β*, they were in the range of -5.4 to -10.1 and  -5.5 to -10.6 kcal/mol, respectively. For COX-2 and COX-1 enzymes binding affinities ranged from -6.8 to -10.1 kcal/mol and from  -5.4 to -10.0 kcal/mol, respectively.

**Table 6:** Binding affinities of the detected metabolites in the alcoholic extract of *F. cretica* L. aerial parts to the active sites of various isoforms of topoisomerase and cyclooxygenase enzymes

*No*.	Metabolite name	Formula	Binding affinity (kcal/mol)				
			Topo I	Topo IIα	Topo IIβ	COX-2	COX-1
1	Linoleic acid	C_18_H_32_O_2_	-5.8	-5.4	-5.5	-6.8	-5.4
2	Isorhamentin 3,7-diglucoside	C_28_H_32_O_17_	-8.5	-9.4	-9.1	-9.2	-8.6
3	*β*-sitosterol	C_29_H_50_O	-7.2	-8.2	-8.4	-7.9	-7.9
4	Atricarpan B	C_18_H_16_O_7_	-7.7	-8.5	-7.9	-8.1	-9.1
5	7*β*-Hydroxy-5-epi-fagonene	C_20_H_34_O_3_	-6.6	-8.0	-7.9	-8.7	-7.2
6	3-*O*-[*β*-D-glucopyranosyl (1–2)-*α*-L-arabinopyranosyl]-27-hydroxy oleanolic acid 28-*O*-[*β*-D-glucopyranosyl (1–6)-*β*-D-glucopyranosyl] ester	C_53_H_86_O_23_	-8.7	-9.6	-9.8	-8.2	-9.2
7	3*β*-(2-*O*-sulfo-R-L-arabinopyranosyl)-27-dihydroxyurs-12-en-28-oic acid 28-*O*-*β*-D-glucopyranoside	C_41_H_66_O_16_S	-8.7	-10.1	-9.0	-8.5	-8.8
8	Geranyllinalool-3-*O*-*β*-D-glucopyranoside	C_26_H_44_O_6_	-6.1	-7.3	-6.9	-7	-7
9	Oleanolic acid	C_30_H_48_O_3_	-8.3	-9.7	-8.7	-7.8	-8.9
10	Isorhamnetin	C_16_H_12_O_7_	-7.3	-8.4	-8.0	-7.4	-7.4
11	Herbacetin-8-methyl ether (sexangularetin)	C_16_H_12_O_7_	-7.0	-7.6	-6.8	-6.9	-7.5
12	3-*O*-([*β*-D-4-*O*-sulfonylglucopyranosyl-(l-3)]-[*β*-D-xylopyranosyl-(l-2)]-*α*-L-arabinopyranosyl)-ursolic acid-28-*O*-[*β*-D-glucopyranosyl] ester (Indicasaponin D)	C_52_H_84_O_24_S	-9.2	-9.3	-9.1	-8.7	-9.5
13	Oleanolic aldehyde acetate	C_32_H_50_O_3_	-8.4	-9.0	-8.7	-7.9	-8
14	Oleanolic acid 3-*O*-*α*-L-rhamnopyranosyl (1–3)-6'-*O*-methyl-*β*-D-glucuronopyranosyl 28-*O*-*β*-D-glucopyranoside	C_49_H_78_O_18_	-9.1	-10.1	-10.6	-9.5	-10
15	22-Hydroxyursolic acid	C_30_H_48_O_4_	-7.8	-8.5	-9.0	-7.9	-8.6
16	Quinovic acid3-*O*-*β*-D-glucopyranoside	C_36_H_56_O_10_	-8.9	-9.8	-8.6	-8.5	-8.8
17	Isorhamnetin-*α*-3-*O*-L-rhamnoside	C_22_H_22_O_11_	-7.7	-8.6	-8.4	-8	-7.6
18	16-*O*-acetylfagonone	C_22_H_34_O_4_	-7.2	-8.9	-7.9	-7.3	-7.7
19	3-*O*-D-glucopyranosyl-(1→3)-*α*-L-arabinopyranoside oleanolic acid	C_41_H_66_O_12_	-8.5	-9.6	-9.0	-9.6	-8.9
20	Rutin	C_27_H_30_O_16_	-8.5	-9.8	-8.4	-10.1	-9.2
21	3*β*-*O*-[*β*-D-glucopyranosyl (1→2)-*α*-L-arabinopyranosyl] olean-12-en-27-al-28-oic acid 28-*O*-[*β*-D-glucopyranosyl (1→6)-*β*-D-glucopyranosyl] ester	C_53_H_84_O_23_	-9.5	-9.9	-10.0	-9.2	-9.5

## Discussion

The Mediterranean region is one of the most important phytogeographic locations in Egypt, it encompasses about 10% of the higher plants in the world [36]. *F. cretica* L. aerial parts were collected from the Mediterranean coast of Egypt have been investigated for TPC and TFC where the phytochemical results suggested that phenolics and flavonoids could be the main contributors for the detected antioxidant potential of the plant in the current study. Recently, plants have become a good source for new anti-inflammatory compounds. COX-2 and COX-1 are isoenzymes that play a key role in the biosynthesis of prostenoids. COX-1 is constitutive and important in gastric cyto-protection and platelet aggregation, whereas COX-2 is an inducible enzyme that is activated in response to inflammation. On the other hand, excessive production of NO is mediated in certain pathological conditions such as septic shock, neurological disorders, and inflammation. So, inhibition of NO is beneficial in treating such conditions. The alcoholic extract of *F. cretica* was tested for its anti-inflammatory potential *via* assessment of its inhibitory impact on COX enzymes and NO as well, and the results revealed that it is effectively inhibited COX-2 and COX-1 enzymes and inhibited NO, comparing with the standards, celecoxib, indomethacin, and ascorbic acid, respectively.

The alcoholic extract of the plant was evaluated for its cytotoxic activity against three human carcinoma cell lines, and it exhibited good inhibitory activity against MCF-7 cell line, followed by HepG2 and CACO2 compared to doxorubicin. It’s noteworthy that *F. cretica* has been reported previously to have cytotoxic effect using brine shrimp assay, potato disc assay, and DNA damage assay [37]. Lam et al., declared that *F. cretica* aqueous extract manifested anticancer potential on breast cancer cell lines through DNA damage induction [38]. Cytotoxic activity of other *Fagonia* species has been reported, for instance, *F. taeckholmiana* extract demonstrated potent activity against breast carcinoma cell lines [39].

On the molecular level, it has been reported that topoisomerases significant targets for several anticancer drugs since they are nuclear universal enzymes that play a valuable role in transcription, replication, chromosome segregation, and repair of DNA [23]. On the other hand, cell apoptosis mainly occurs because of the activation of caspases as caspase-3, caspase-8, and caspase-9. Caspase-9 is one of the caspase motivators which is responsible for activation of caspase-3 [25]. Hence, the alcoholic extract of *F. cretica* plant was evaluated for its inhibitory effect on topo I enzyme using breast carcinoma (MCF-7) cell line and for the stimulation of apoptosis through caspase-9 assay in MCF-7 cell line, compared to doxorubicin as standard, and results showed inhibition of topo I and induction of caspase-9 levels as well. It may be concluded that *F. cretica* could afford anticancer effect *via* inhibition of Topo I enzyme and induction of caspase dependent pathway.

Studying the metabolic profiling results of the alcoholic extract of *F. cretica* revealed molecular ion peak at *m/z* 280.1368 corresponding to the molecular formula C_18_H_32_O_2_ which was identified as linoleic acid, previously detected in *F. cretica* [40]. Ten triterpenoid saponin compounds with molecular ion peaks at *m/z* 1090.6401, 846.4536, 456.2779, 1124.8413, 482.3746, 954.6166, 472.2744, 648.3759, 750.4486, and 1088.6292 were in consistent with the molecular formulas C_53_H_86_O_23_, C_41_H_66_O_16_S, C_30_H_48_O_3_, C_52_H_84_O_24_S, C_32_H_50_O_3_, C_49_H_78_O_18_, C_30_H_48_O_4_, C_36_H_56_O_10_, C_41_H_66_O_12_, and C_53_H_84_O_23_, respectively. These triterpenes were identified as, 3-*O*-[*β*-D-glucopyranosyl (1–2)-*α*-L-arabinopyranosyl] 27-hydroxy oleanolic acid 28-*O*-[*β*-D-glucopyranosyl (1–6)-*β*-D-glucopyranosyl] ester (6), 3*β*-(2-*O*-sulfo-R-L-arabinopyranosyl)-27-dihydroxyurs-12-en-28-oic acid 28-*O*-*β*-D-glucopyranoside (7), oleanolic acid (9), indicasaponin D (12), oleanolic aldehyde acetate (13), oleanolic acid 3-*O*-*α*-L-rhamnopyranosyl (1–3)-6'-*O*-methyl-*β*-D-glucuronopyranosyl 28-*O*-*β*-D-glucopyranoside (14); 22-hydroxyursolic acid (15), quinovic acid 3-*O*-*β*-D-glucopyranoside (16), 3-*O*-D-glucopyranosyl-(1→3)-*α*-L-arabinopyranoside oleanolic acid (19), 3*β*-*O*-[*β*-D-glucopyranosyl (1→2)-*α*-L-arabinopyranosyl] olean-12-en-27-al-28-oic acid 28-*O*-[*β*-D-glucopyranosyl (1→6)-*β*-D-glucopyranosyl] ester (20), respectively. 3*β*-(2-*O*-sulfo-*α*-L-arabinopyranosyl)-27-dihydroxyurs-12-en-28-oic acid 28-*O*-*β*-D-glucopyranoside (7) and 3-*O*-D-glucopyranosyl-(1→3)-*α*-L-arabinopyranoside oleanolic acid (19) were isolated from *F. arabica* [41, 42], indicasaponin D (12) was previously isolated from *F. indica* [43], oleanolic acid 3-*O*-*α*-L-rhamnopyranosyl (1–3)-6'-*O*-methyl-*β*-D-glucuronopyranosyl 28-*O*-*β*-D-glucopyranoside (14) has been isolated from *F. mollis* [44] and finally other triterpenes were previously isolated from the present species *F. cretica* [40, 45]. Four flavonoids with characterized ion peaks at *m/z* 640.4577, 316.1950, 462.3607, and 610.048 corresponding to the molecular formulas C_28_H_32_O_17_, C_16_H_12_O_7_, C_22_H_22_O_11_, and C_27_H_30_O_16_ were annotated as isorhamnetin 3,7-diglucoside (2), isorhamnetin (10) or herbacetin-8-methyl ether (sexangularetin) (11), isorhamnetin-*α*-3-*O*-L-rhamnoside (17) and rutin (20), respectively. Isorhamnetin (10) or herbacetin-8-methyl ether (sexangularetin) (11) were identified in *F. mollis* Del var. Grandiflora [46], while rutin (20) was previously isolated from *F. cretica* [47], and the other two flavonoids were formerly isolated from *F. thebaica* [48]. Additional mass ion peak at *m/z* 414.2129 with the suggested formula C_29_H_50_O was dereplicated as *β*-sitosterol (3), which previously detected in *F. indica* [49, 50]. The pterocarpan atricarpan B (4), belongs to the second largest group of natural isoflavonoids that plays a significant role as a phytoalexin and corresponds to mass ion peak at *m/z* 344.0979 and suggested molecular formula C_18_H_16_O_7_ was previously isolated from *Zygophyllum eurypterum* [51]. Moreover, three diterpenes, 7*β*-hydroxy-5-epi-fagonene (5), geranyllinalool-3-*O*-*β*-D-glucopyranoside (8) with the identified formulas C_20_H_34_O_3_ and C_26_H_44_O_6_ predicted for mass ion peaks with *m/z* 322.0995 and 452.3227 which were isolated earlier from *F. mollis* [52] as well 16-*O*-acetylfagonone (18) an erythroxan-type diterpene that has been identified with the corresponding formula C_22_H_34_O_4_, predicted for mass ion peak with *m/z* 362.3102 that was isolated earlier from and *F. bruguieri* [53].

Results of the current work suggest anticancer activity of *F. cretica* extract. Besides, a number of previous researches reported the anticancer potential of *F. cretica* plant [37, 38] and most of previous chemical investigations of the plant reported the isolation of saponin compounds [40, 45, 54] where some of these reports confirmed the anticarcinogenic potential of the isolated triterpenoid saponins [55]. These results along with our current metabolic profiling study that detected various saponins in *F. cretica* extract motivated us to prepare saponin-rich fraction of the plant and to explore its effect on the apoptotic markers under study (topo I and caspase 9) in comparison with the non-saponin fraction of the extract. Results unveiled similar effects exerted by both fractions on topo I and caspase 9, indicating that the anticancer potential of the plant is not inclusively due to saponin content, but other constituents contributed likewise to the activity, and they all act synergistically as cytotoxic agents.

For in depth exploration of binding mechanism of the individual *F. cretica* metabolites into cancer markers, molecular docking experiments were conducted on the metabolites identified in *F. cretica* extract targeting the active sites of key proteins involved in cancer progress (topo I, topo II*α*, and topo II*β*) and additionally those involved in inflammation mechanism (COX-2 and COX-1). Results of the investigation unveiled high binding affinities of almost all the detected metabolites to active sites of the enzymes under study.

Topoisomerases and their proper performance are one of the essential factors in many cellular processes. The augmented topoisomerases activity detected in various cancers resulted in the selective action of the agents that act as topoisomerase inhibitors [56]. Topo I is a monomer protein, its expression is constant throughout various cell cycle phases, while topo II isoenzymes are homodimers; the expression of topo II*α* depends on the stage of the cell cycle where it is not detected in G_0_ phase and increases during other phases in the contrary to topo II*β* that remains stable throughout the whole cell cycle [57]. Docking outcomes showed that almost all the metabolites under investigation exhibited good binding affinities to the three forms of topoisomerases (I, II*α*, and II*β*). For Topo I, six saponins (21, 12, 14, 16, 7, and 6) showed the highest binding affinities (-9.5, -9.2, -9.1, -8.9, -8.7, and  -8.7 kcal/mol, respectively) even higher than the co-crystallized ligand camptothecin (-8.6 kcal/mol). For the purpose of simplicity, interactions of compound 21 will be highlighted where docking investigation revealed hydrogen bonds with Lys532 that has been delineated to be essential for the transient state stabilization and as a general acid catalyst during cleavage of the substrate DNA [58]. Another hydrogen bonding with the conserved active site residue Arg488 which plays an imperative role in the nicking-closing processes on the substrate DNA [59]. Furthermore, hydrogen bonds with Asn352, Lys443, Val502, and Lys751, carbon-hydrogen bond with Lys493, and *π*-alkyl interaction with Phe723 have been detected. Among non-saponin metabolites, rutin (20) and isorhamnetin 3,7-diglucoside (2) produced the highest binding affinity score for topo I (-8.5 kcal/mol) where rutin interacted *via* four hydrogen bonds with Asn491, Lys493, Thr501, and Lys532, carbon hydrogen bond with Arg488, π-σ interaction with Thr498, π-alkyl interactions with Arg364 and Lys532, and electrostatic interactions with Lys493 and Asp533. The saponin compund No. (14) exhibited the highest binding affinities to both topo II*α* and topo II*β* (-10.1 and -10.6 kcal/mol, respectively) that are comparable to those of the co-crystallized ligand; etoposide (-12.3 and -11.0 kcal/mol, respectively) where it lodged itself in the binding sites of both enzymes via hydrogen bond interactions with Sr763 and Arg929 in topo II*α* and Lys739, Asp726, and Thr874 in topo II*β*. For non-saponins, rutin exhibited the top binding affinity to topo II*α* (-9.7 kcal/mol) with five hydrogen bonds to Ser709, Leu722, His759, Glu837, and Glu839, three alkyl interactions with Arg713 and Pro725, π-alkyl interaction with Pro725, carbon- hydrogen bonds with Ser717 and Pro724, π- π stacking with Phe1003, and electrostatic interactions with Arg673 and Glu712, while isoramnetin-3,7-diglucoside (2) manifested the highest binding affinity to topo II*β*, a variety of interactions were observed including seven hydrogen bonds with Arg743, Ala772, Glu853, His1021, and Gly1023, three carbon- hydrogen bonds with Lys744, His774, and Glu855, three π-alkyl interactions with Pro740 and Leu845, π- π T-shaped interaction with Phe1019, and electrostatic interaction with Glu728.

For COX-2, the flavonoid rutin demonstrated the highest binding affinity (-10.0 kcal/mol) which is higher than the co-crystallized ligand (flufenamic acid, -8.8 kcal/mol) and even higher than the standard celecoxib (-9.9 kcal/mol). Rutin has been reported previously to inhibit COX-2 enzyme [60]. Following rutin (20) are the two saponins compounds (19 and 14) with binding affinities of -9.6 and -9.5 kcal/mol, respectively to the COX-2 active site, it is worthy to note that the two metabolites have not been tested for COX inhibition previously. Compound (19) bonded in the COX-2 active site with the aglycone part located near the hydrophobic side pocket and formed various alkyl and *π*-alkyl interactions with Val89, Leu93, Phe107, Ala111, Ile112, and Tyr115, while the sugar part exhibited two hydrogen bonds with Phe107 and Ser114 amino acids and a carbon-hydrogen bond with Ala111. On the other hand, compound (14) lodged itself in a hydrophilic pocket via hydrogen bonds with Thr94, Gln192, Glu346, Gly354, and Ala562, carbon hydrogen bonds with Asp347, Gly354, and Pro514, and π-alkyl interactions with His356 and Phe580.

## Conclusion

Phytochemical analysis of the alcoholic extract of the Egyptian desert plant *Fagonia cretica* L. (Family: Zygophyllaceae) revealed total phenolic (TPC) and total flavonoid (TFC) contents 2.4 ± 0.12 mg GAE/g and 0.18 ± 0.01 mg RE/g, respectively. Whereas biological investigation demonstrated in vitro antioxidant potential *via* DPPH assay as 1.4 ± 0.1 mg AEAC/g and in vitro anti-inflammatory activity through inhibition of COX-2 and COX-1 with IC_50_ values of 13.02 ± 0.61 and 26.51 ± 0.83 µg/ml, respectively and inhibition of nitric oxide with IC_50_ of 147.05 ± 9.61 µg/ml. Furthermore, cytotoxic activity was carried out against MCF-7, HepG2, and CACO2 cell line with IC_50_ values of 6.9 ± 0.53, 7.6 ± 0.42, and 9.2 ± 0.35 µg/ml, respectively, which was supported by in vitro topoisomerase I inhibition (IC_50_ = 13.57 ± 0.71 µg/ml) and caspase 9 induction by 5.66 folds. Metabolic profiling using LC-HRMS technique resulted in dereplication of 21 compounds including triterpenoid saponins, flavonoids, diterpenoids, etc. Considering these results and in view of that saponins were the major detected and isolated constituents from *Fagonia* species, a comparison of anticancer potentials of saponin-rich fraction and non-saponin fraction of the plant was conducted where they showed comparable effects on topoisomerase I and caspase 9 enzymes indicating that all the metabolites may act synergistically to afford the anticancer influence. From this perspective, in-depth molecular docking investigation unveiled high binding affinities of almost all the detected metabolites to the active sites of topo I, II*α*, and II*β* enzymes and COX-2 and COX-1 as well. These results shed light on *Fagonia cretica* as a potential herbal drug for further investigation.

## Data Availability

All data generated or analyzed during this study are included within the manuscript.
